# Effects of Arginine Concentration on the *In Vitro* Expression of Casein and mTOR Pathway Related Genes in Mammary Epithelial Cells from Dairy Cattle

**DOI:** 10.1371/journal.pone.0095985

**Published:** 2014-05-01

**Authors:** Mengzhi Wang, Bolin Xu, Hongrong Wang, Dengpan Bu, Jiaqi Wang, Juan-Jose Loor

**Affiliations:** 1 College of Animal Science and Technology, Yangzhou University, Yangzhou, Jiangsu Province, P.R. China; 2 State Key Laboratory of Animal Nutrition, Institute of Animal Science, Chinese Academy of Agricultural Sciences, Beijing, P.R. China; 3 Mammalian NutriPhysioGenomics, Department of Animal Sciences and Division of Nutritional Sciences, University of Illinois, Urbana, Illinois, United States of America; State Key Laboratory of Pathogen and Biosecurity, Beijing Institute of Microbiology and Epidemiology, China

## Abstract

Arginine (Arg) is a conditionally-essential amino acid that is taken up by bovine mammary gland in excess of its output in milk. In this study we evaluated the effects of Arg concentration on the expression of casein and signaling pathway-related genes in mammary epithelial cells. The treatments (applied for 24 h) were designed to be devoid of Arg 0X (control; 0.00 mg/L), resemble the profile of Arg in casein (Arg 1X; 278.00 mg/L), be deficient [Arg 0.25X (69.50 mg/L) and Arg 0.5X (139.00 mg/L)], or be in excess of the amount in casein [Arg 2X (556.00 mg/L), Arg 4X (1,112 mg/L), and Arg 8X (2,224 mg/L)]. The expression of CSN1S, CSN3 and mTOR in the experimental groups was higher than those of the control group (*P*<0.05). Except for Arg 0.25X and Arg 8X (*P*>0.05), the expression of CSN1S2, CSN2 and JAK2 in other experimental groups was higher (*P*<0.05) than those in the control group. Except for Arg 8X (*P*>0.05), the expression of STAT5 in the other experimental groups was higher than those of the control (*P*<0.05). It also was observed that except for Arg 0.5X, the S6K expression was higher in other experimental groups than the control (*P*<0.05). In contrast, except for Arg 0.25X the other experimental groups resulted in lower 4EBP1 expression than the control (*P*<0.05). Among groups, the expression of CSN1S1, CSN1S2, CSN2, CSN3, JAK2, STAT5, mTOR and S6K gene was highest with Arg 2X (*P*<0.05); the reverse was true for 4EBP1 gene, with the lowest expression in this group (*P*<0.05). Taken together, Arg appears to play an important role in the transcriptional regulation of casein genes and mTOR-related genes in bovine mammary epithelial cells.

## Introduction

There is growing recognition that besides their role as building blocks of proteins and polypeptides, some amino acids (AA) regulate key metabolic pathways that are necessary for maintenance, growth, reproduction, and immunity [1]. Arginine, as a AA, plays an important role in the control of cell division, wound healing, removal of ammonia from the body, protein synthesis, and the release of hormones [2], [3], [4]. Interestingly, reviews of the literature in dairy ruminants concluded that the uptake of arginine by the mammary gland greatly exceeded its output in milk [5], [6]. Except for its use during milk protein synthesis, those reviews concluded that the extra arginine might exert other unknown biological or metabolic functions in the mammary gland.

The availability of AA in the lactating cow is critical for mammary protein synthesis [7]. Previous work on AA metabolism in dairy cattle have focused on achieving an optimal balance in the diet to provide the required precursors for maintaining and enhancing milk protein synthesis [5], [8]. Recently, Rius *et al.* [9] investigated signaling pathways responsive to casein and starch infusion in primiparous mid-lactation Holstein cows. Results revealed that cell signaling molecules involved in the regulation of milk protein synthesis responded differently to the various nutritional stimuli. Subsequent work by Appuhamy *et al.* [10] revealed regulatory roles of essential AA other than arginine in mammary cell protein synthesis.

Research in monogastrics has underscored the importance of arginine in other biological functions, and recently the function and the metabolism of arginine in ruminant mammary gland was re-considered by Doepel and Lapierre [11]. Using abomasal infusions to increase arginine availability in Holstein cows, they evaluated if the supply of arginine relative to controls would alter milk and milk protein yield. Their results revealed that milk protein yield was increased by infusion of arginine compared with a control that included infusion of water. In their work, the Arg infusion had similar effects as other EAA infusion, this might be because Arg can be converted from other EAA in body. But the research also revealed that the range in the ratio of Arg uptake to output was between 2.52 to 2.12 and was still largely in excess of Arg secretion in milk protein [11]. Despite the positive result with arginine infusion, the functions and potential mechanisms of excess arginine uptake by mammary gland of the lactating cow remain unclear.

A compelling case was recently made for more in-depth studies of molecular regulation of lactation, particularly in livestock species [12], and specifically on the effect of mTOR in the regulation of milk protein synthesis [13]. Our previous work also demonstrated that arginine increases casein protein synthesis in bovine mammary epithelial cells [14], thus, we hypothesized that arginine might partly regulate milk protein synthesis through alterations in expression of genes related with the mTOR and STAT signaling pathways. The specific objective was to elucidate the effect of arginine on mRNA expression of casein, mTOR, and STAT genes in bovine mammary epithelial cells.

## Materials and Methods

### Animal and Mammary Tissue Sampling

Three multiparous healthy lactating Chinese Holstein cows at similar lactation stages (day 100±5 of lactation) were obtained from the Experimental Farm of Inner Mongolia Agricultural University. The cows were sacrificed within 1 h after milking. Immediately before exsanguination, milked mammary glands were split down the mid-line, and mammary tissue was excised from the center portion of each of the four quarters. Tissue samples from four quarters of the mammary glands of each cow were mixed and processed together. After removing obvious pieces of connective tissue and fat, the mammary tissues were cut into small pieces of 0.5 mm in thickness [15]. The tissue pieces from each cow were divided into 6 parts and placed in DMEM/F12 medium supplemented with the antibiotic-antimycotic mix (Sigma-A5955) as described by Chen *et al.* [14]. The study was approved by the Inner Mongolia Agricultural University Animal Care and Use Committee.

### Tissue Incubation and Purification of Mammary Epithelial Cells

The tissue culture and the mammary epithelial cell isolation were carried out according to the methods described by Wu [16], O’Quinn *et al.* [15] and Xu *et al.* [17]. The method for isolation and purification of mammary cells is described in detail below. Firstly, collagenase digestion was used to isolate the mammary cells from tissue. An equal volume of collagenase (Type II, Gibco 17101-015) was added into 5 ml tube (the working solution is 0.5%) containing tissue slices, placed in a constant temperature shaker at 37°C for 1 h, and then filtered through 80 mesh prior to collecting cells by centrifugation (1,500 r/min for 5 min at room temperature). Cells were then added to 25-mL polypropylene flasks containing DMEM/F12 medium and placed in an incubator at 37°C. When cells reached 90% confluence, 0.25% Trypsin was added into the culture to purify epithelial cells from fibroblast according to their different sensitivity to Trypsin.

**Figure 1:**
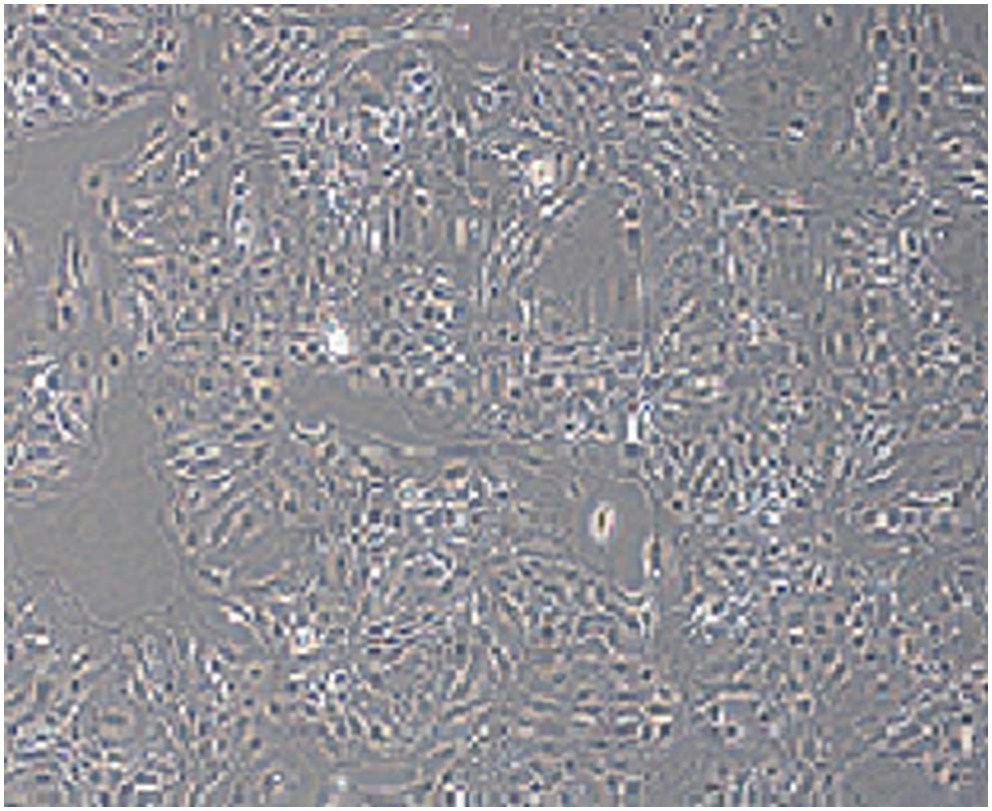
Microscope image of F2 mammary epithelial Cells (×100).

**Figure 2:**
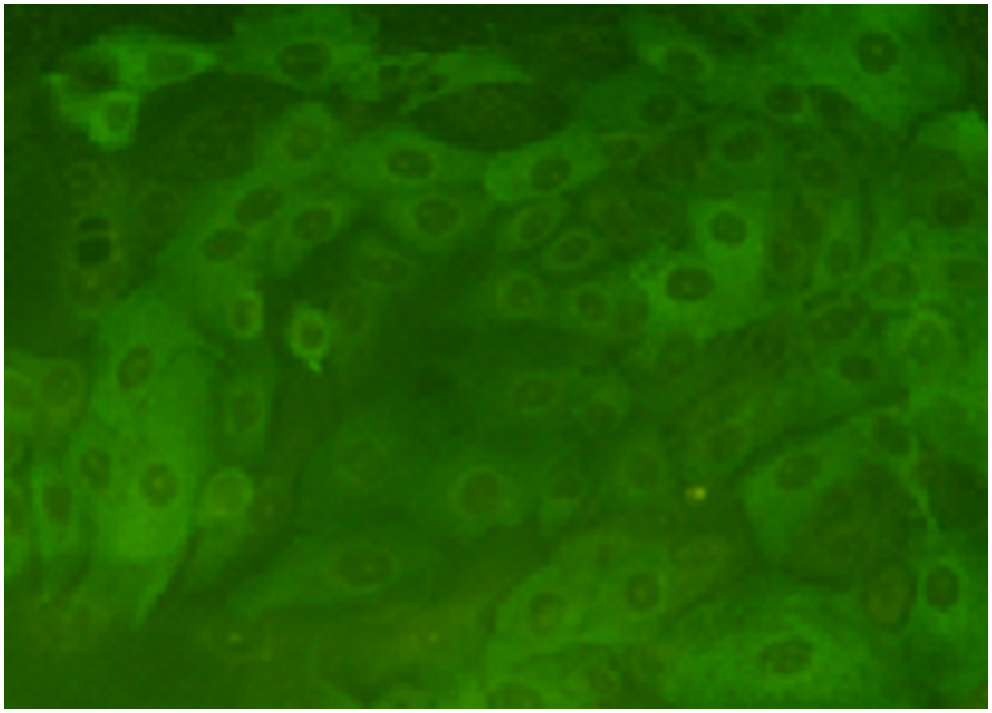
Immunofluorescence of Cytokeratin 18 (CK-18) (×200).

The isolated mammary epithelial cells were evaluated by morphology and immunofluorescence cell staining with fluoresceinisothiocyanate. The antibodies used in immunofluorescence were CK18 mouse monoclonal antibody (Sigma-Aldrich: SAB3300015-100UL) as first antibody and goat anti-mouse IgG conjugated FITC (Sigma-Aldrich: F5387) as secondary antibody following the manufacturer’s protocol. From figure 1 it can be seen that the purified cells had the characteristic cobblestone morphology of epithelial cells, and there were clear boundaries between cells but with close connections. Additionally, cell monolayer growth showed visible dome structures. From figure 2 it can be seen that the expression of CK-18 was positive in purified mammary epithelial cells. Lastly, the purified epithelial cells were mixed and incubated at 5×10^5^/mL (counting by Cytorecon CYT-1000) on plastic dishes, and the 2^nd^ generation cells were used for the arginine study.

### Preparation of the Arginine-removal Basic Medium

As arginine exists in commercial cell medium, a medium without arginine had to be prepared taking into account the composition of DMEM/F12 (Gibco, Invitrogen, Catalog #11320082, Life Technologies Corporation, US). The basic medium devoid of arginine was prepared by combining the individual reagents (according to the composition of DMEM/F12 in ultrapure water). The basic medium was used in the transition process immediately before the arginine study. The AA profile of this arginine-removal basic medium was described in our previous work [14]; for the experimental treatments the arginine was added into the medium in accordance with the experimental design described below.

### Experimental Design and Treatments

A single factorial experimental design varying the arginine levels was used to achieve the specific treatment groups. The target response that we focused on was casein production, thus, we attempted to develop media with combinations of AA closely resembling the profile in casein. The media containing arginine that most-closely resembled the profile of casein was defined as Arg 1X. The other treatment groups were made simply by varying the arginine concentration and without changing the concentration of other AA. The design included 7 groups, the same as our previous work [14]: Arg 0.25X (arginine at 69.50 mg/L), Arg 0.5X (arginine at 139.00 mg/L), Arg 1X (arginine at 278.00 mg/L), Arg 2X (arginine at 556.00 mg/L), Arg 4X (arginine at 1,112 mg/L), Arg 8X (arginine at 2, 224 mg/L) as experimental groups, and Arg 0X (arginine at 0.00 mg/L) was set as the control group (without arginine in the medium). Each treatment was run in triplicate, and a set of appropriate blanks (without cell incubation) was included.

### RNA Extraction from Mammary Cells

After culture for 24 hours the reactions were stopped by removing medium and collecting cells. Cells were then frozen in liquid nitrogen prior to storage at −80°C. The RNA was extracted and purified using a cellular RNA extraction kit (DP430 spin column kit, Tiangen Biotech, Beijing, China). The extraction steps were carried out following the manufacturer's protocol. RNA quantity and integrity were confirmed with a NanoDrop 1000 spectrophotometer (NanoDrop Technologies, Wilmington, DE, US).

### Measurement of Target Gene Expression

RNA samples were reverse-transcribed using PrimeScript 1st Strand cDNA Synthesis Kit (TaKaRa Code: D6110A, TaKaRa Biotechnology (Dalian) Co., Ltd., Dalian, China), according to the manufacturer’s instructions. The real time-PCR was performed in a Bio-Rad IQ5 Real-Time PCR (Bio-Rad Laboratories, Inc. Hercules, CA, US), using SYBR Premix Ex Taq II kit (TaKaRa Code: DRR081A, TaKaRa Biotechnology (Dalian) Co., Ltd., Dalian, China). All reactions were run using the protocol below: 30 s at 95°C; 10 s at 95°C, 20 s at annealing temperature, and 30 s at 72°C for 40 cycles. The same conditions were performed on an equal amount of RNAase-free water as a negative control. The information of primers for target and internal reference gene were displayed in table 1 including the glyceraldehyde-3-phosphate dehydrogenase (GAPDH) as internal control gene. It is recognized that using a single ICG is not ideal [18] but GAPDH has been used previously as the sole ICG in studies of mammary cell gene expression.

**Table 1:** The information of real time PCR primers of target genes detected in mammary epithelial cell from dairy cattle *by in vitro* culture with different arginine levels.

Items	Sequences of primers (5′ –3′)	Genebank ID	Production	Annealing temperature
CSN1S2 (αs2-casein)	F:AGCTCTCCACCAGTGAGGAA	NM_174528.2	150 bp	56°C
	R:GCAAGGCGAATTTCTGGTAA			
CSN3 (kappa-casein)	F:CCAGGAGCAAAACCAAGAAC	NM_174294	148 bp	56°C
	R:TGCAACTGGTTTCTGTTGGT			
JAK2 (Janus kinase 2)	F:ACAGGGGCTGGCGTTCA	XM_002689603.1	146 bp	61°C
	R:TATTGGTAACCAACAGCTCAAGG			
mTOR (Mechanistic target of rapamycin)	F:ATGCTGTCCCTGGTCCTTATG	XM_001788228.1	199 bp	63°C
	R:GGGTCAGAGAGTGGCCTTCAA			
STAT5 (Signal transducer and activatorof transcription 5)	F:AAGACCCAGACCAAGTTCGC	NM_001012673.1	203 bp	64°C
	R:AGCACCGTGGCAGTAGCAT			
S6K (Ribosomal protein S6 kinase)	F:GGACATGGCAGGGGTGTTT	NM_205816.1	162 bp	58°C
	R:GGTATTTGCTCCTGTTACTTTTCG			
4EBP1 (4E binding protein 1)	F:GGCAGGCGGTGAAGAGTC	BC120290.1	177 bp	60°C
	R:CCTGGGCTGCGGGAT			
GAPDH (glyceraldehyde-3-phosphate dehydrogenase)	F:GGGTCATCATCTCTGCACCT	XM_001252479	177 bp	59°C
	R:GGTCATAAGTCCCTCCACGA			

### Statistical Analysis

The statistical analysis was carried out by one-way analysis of variance (ANOVA) followed by the Duncan’s test for post-hoc multiple comparisons of treatment means using SAS software (SAS Inst., Cary, NC, USA). In addition, a regression analysis was conducted using SPSS (v16.0). The random effect in the ANOVA model was replicate and the fixed effect was level of arginine. Significance was declared at *P* <0.05.

## Results

### Effects of Arginine Level on the Expressions of Casein Genes

**Figure 3:**
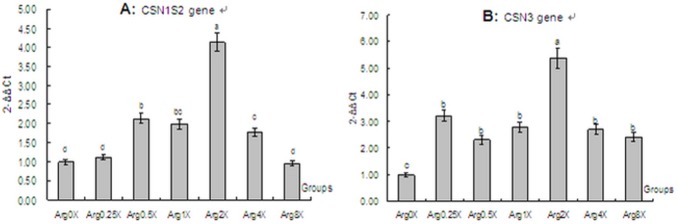
*In vitro* effect of arginine level on the expression of casein genes in mammary epithelial cells from lactating dairy cattle. And: Panel A, CSN1S2 gene; and Panel B, CSN3 gene. Arg 0X, control without supplemental Arg (0.00 mg/L); Arg 0.25X, deficiency in casein (69.50 mg/L); Arg 0.5X, deficiency in casein (139.00 mg/L); Arg 1X, to match its concentration in casein (278.00 mg/L); Arg 2X, excess in casein (556.00 mg/L); Arg 4X, excess in casein (1,112 mg/L); and Arg 8X, excess in casein (2,224 mg/L). Different letters indicate significant difference between means (*P*<0.05).

Figure 3A/B shows the relative expression of Casein alpha s2 (CSN1S2) and Casein kappa (CSN3). The relative expression of Casein alpha s1 (CSN1S1) and Casein beta (CSN2) has been published previously (Chen *et al.*, 2013). Expression of CSN1S2 and CSN3 in the Arg 2X (*P*<0.05) (containing 556.00 mg/L arginine in culture medium) was higher than other treatments and the control. Except for CSN1S2 in the Arg 0.25X and the Arg 8X, all other Arg concentrations resulted in greater expression of these genes compared with the control group (*P*<0.05).

The relative expression of CSN1S2and CSN3 increased from the Arg 0.25X to the Arg 2X; however, expression decreased when comparing the Arg 4X to the Arg 8X.

**Figure 4:**
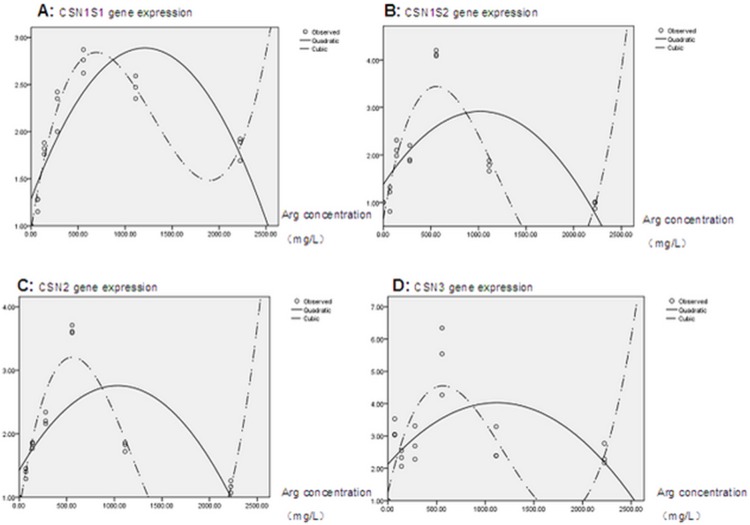
Cubic regression analysis of casein gene expression and arginine concentration. Panel A, CSN1S1 (data cited from Chen *et al.* (2013)); Panel B, CSN1S2; Panel C, CSN2 (data cited from Chen *et al.* (2013)); and Panel D, CSN3 gene.

The quadratic and cubic regression analysis results for casein gene expression (see figure 4A–D, data of CSN1S1 and CSN2 were cited from the previous work by Chen *et al.* [14]) showed that, compared with a quadratic regression equation, a cubic regression equation had higher adjusted coefficient of determination (adjusted R^2^). The cubic equations for casein genes described below were used in this work to evaluate gene expression.

CSN1S1,
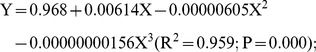



CSN1S2,
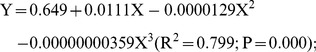



CSN2,
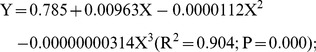



CSN3,
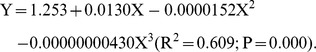



According to these equations, the calculated highest expression for casein genes was 2.37, 2.81, 2.66, and 3.76 for CSN1S1, CSN1S2, CSN2, CSN3, respectively; the corresponding arginine concentration was 434.44 mg/L, 372.36 mg/L, 371.784 mg/L, and 369.65 mg/L.

### Effect of Arginine Level on the Expression of Signaling Pathway Related Genes

**Figure 5:**
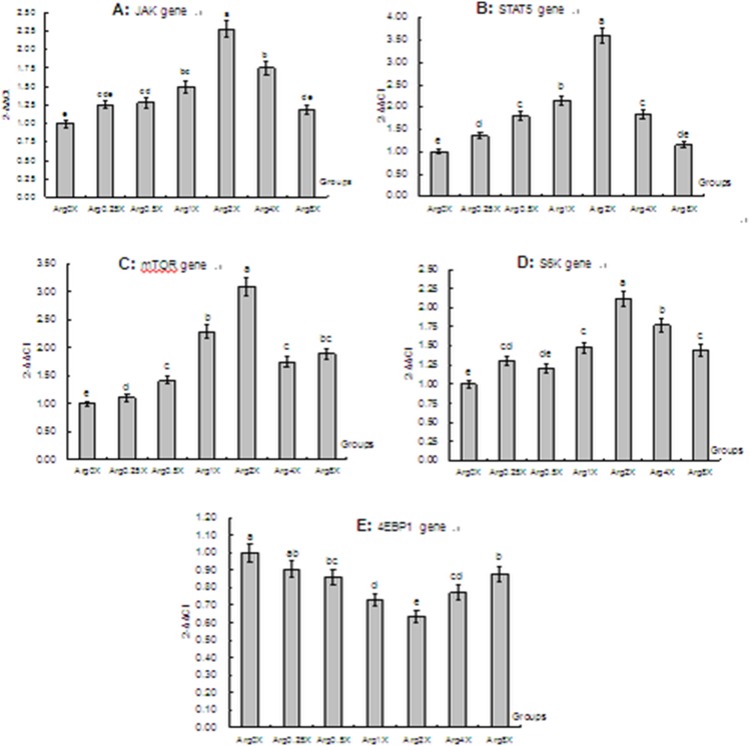
Effects of arginine level on the expressions of signaling pathways related genes in mammary epithelial cells from lactating dairy cattle *in vitro*. Panel A, JAK2; Panel B, STAT5; Panel C, mTOR; Panel D, S6K; and Panel E, 4EBP1 gene. Arg 0X, control without supplemental Arg (0.00 mg/L); Arg 0.25X, deficiency in casein (69.50 mg/L); Arg 0.5X, deficiency in casein (139.00 mg/L); Arg 1X, to match its concentration in casein (278.00 mg/L); Arg 2X, excess in casein (556.00 mg/L); Arg 4X, excess in casein (1,112 mg/L); and Arg 8X, excess in casein (2,224 mg/L). Different letters indicate significant difference between means (*P* < 0.05).

The results of the expression of signaling pathway related genes are presented in figure 5A–E. Similar to results of the casein genes, the relative expression of Janus kinase 2 (JAK2), Signal transducer and activator of transcription 5 (STAT5), Mechanistic target of rapamycin (mTOR), and Ribosomal protein S6 kinase (S6K) was highest in the Arg 2X (*P*<0.05) compared with other doses and the control group. In addition, the pattern of change in the expression of these genes was similar to the casein genes, with a gradual increase up to Arg 2X followed by a decrease as the concentration of arginine increases. The opposite response to Arg dose was observed for the expression of Eukaryotic translation initiation factor 4E binding protein 1 (EIF4EBP1), with Arg 2X leading to the lowest expression among the groups (*P*<0.05); additionally, except for the Arg 0.25X, the other experimental groups led to lower 4EBP1 expression than the control group (*P*<0.05).

**Figure 6:**
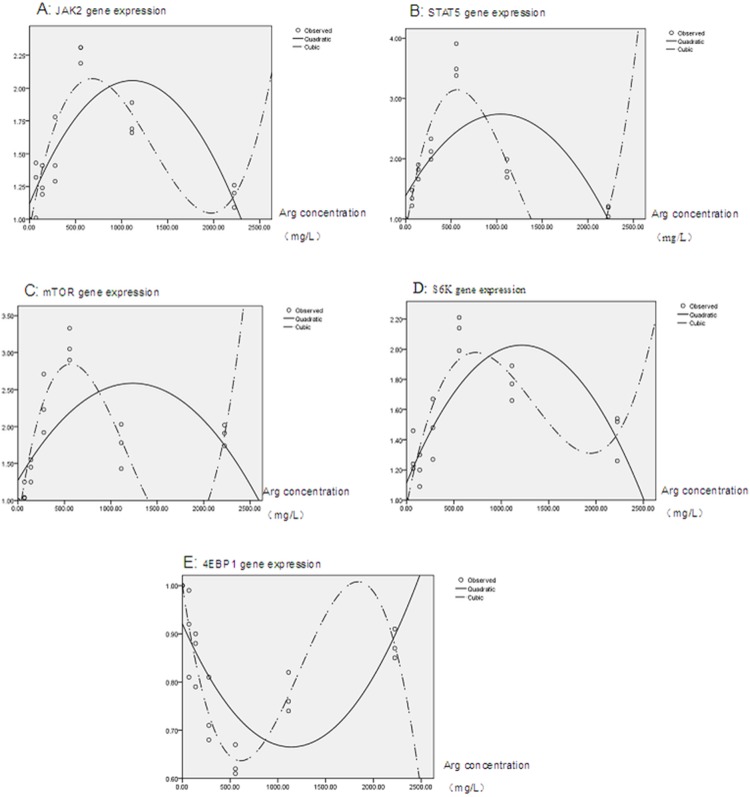
Cubic regression analysis of signaling parthway related gene expression and arginine concentration. Panel A, JAK2; Panel B, STAT5; Panel C, mTOR; Panel D, S6K; and Panel E, 4EBP1.

Quadratic and cubic regression analysis results (see figure 6A–E) of signaling pathway related gene showed that, a cubic regression equation (compared with the quadratic regression equation) with higher adjusted R^2^ provided a better fit to each set of observations. The cubic equations for signaling pathway related genes below were used in this work to evaluate gene expression.

JAK2,
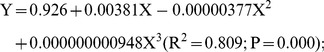



STAT5,
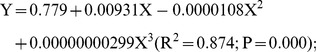



mTOR,
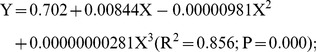



S6K,
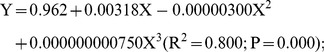



4EBP1,
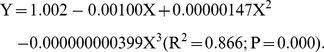



According to these equations, the calculated highest expression for signaling pathway related genes was 2.072, 3.131, 2.846, and 1.976 for JAK2, STAT5, mTOR, and S6K, respectively; the corresponding arginine concentration was 679.42 mg/L, 562.34 mg/L, 569.55 mg/L, and 729.64 mg/L. The minimum expression was 0.81 and the corresponding arginine concentration was 407.87 mg/L for 4EBP1 gene.

## Discussion

A number of studies in monogastric animals have revealed that arginine plays several key functions including serving as precursor for the synthesis of nitric oxide and decreasing blood pressure [19], enhancing cell division [20], [21], and inducing the release of hormones and promoting protein and DNA synthesis [22]. Our preliminary *in vitro* studies showed that arginine increased the synthesis of casein in mammary epithelial cells from dairy cattle. Those results were substantiated by the fact that casein protein, including αs-casein and β-casein, in cultures with Arg 2X was the highest among the groups. For example, αs-casein and β-casein concentration due to Arg 2X increased by 120% and 96% compared with Arg 0X, by 64% and 19% compared with Arg 1X, by 141% and 24% compared with 4X, and by 152% and 53% compared with 8X [14]. In the present study, we attempted to find potential gene transcription mechanisms of Arg stimulation of casein synthesis.

In general, as a key previous step in protein expression, there is a correlation between gene transcription (e.g. mRNA expression) and protein expression [23]. We speculate that the observed stimulatory effects of Arg on casein expression (up to 2X the basal concentration) was associated at least in part with alterations, direct or indirect, with mRNA transcription of the casein genes. The results of casein gene expression in the current work confirmed our speculation that an “optimal” concentration of arginine in culture medium could increase casein gene expression in mammary epithelial cells.

From the multi-comparison ANOVA analysis we observed that the “optimal” concentration of arginine was 556.00 mg/L; further cubic regression analysis indicated that the “optimal” concentration of arginine for upregulation of casein gene expression varied depending on the specific casein gene (CSN1S1, CSN1S2, CSN2, and CSN3), but within a small range from 369.65 mg/L to 434.44 mg/L in culture medium. The stimulatory effects of Arg on casein expression were also associated with greater cell proliferation/growth as reported previously by our group [17].

Several transcription factors control expression of the major milk proteins, particularly the caseins [24]. The casein genes are all present on one chromosome (e.g., chromosome 6 for bovine and chromosome 5 for mouse) and in a cluster of single-copy genes [25]. Previous reports confirmed that casein gene transcription is mediated by Stat5, a member of a transcription factor family that transmits signals from cytokine and growth factor receptors to nuclear target genes [26]. While Stat5 is phosphorylated by JAK2 on a conserved tyrosine (Y694 or Y699, depending on species), resulting in its dimerization, translocation to the nucleus, and binding to a specific sequence [27].

Nutrients and hormones may modulate mammary protein synthesis through the mTOR signaling pathway [28]. In the bovine, STAT5 responds to prolactin and other lactogenic growth factors and its activity increases during lactation mostly due to phosphorylation [29]. Subsequently, Toerien *et al.* [30] showed that infusion of essential AA (EAA) plus glucose reduced phosphorylation of the IRS target eukaryotic initiation factor (eIF) 2 in mammary tissue and increased phosphorylation of the mTOR targets, ribosomal S6 kinase 1 (S6K1) and S6. Wu [1] concluded that functional AA such as arginine, cysteine, glutamine, leucine, act not only as cell signaling molecules but also as regulators of gene expression and the protein phosphorylation cascade. Increasing evidence suggests that arginine might directly activate anabolic cellular signaling pathways [31], [32].

The increase in expression of JAK2, STAT5, mTOR and S6K with the Arg 2X group (556.00 mg/L in culture medium) in this study might suggest a stimulation of protein synthesis [23], [28] as reported in previous work cited above. In the same fashion, the downregulation of 4EBP1 agreed with the negative role of 4EBP1 on protein translation [33], [34], a process that consequently stimulates protein synthesis. The cubic regression analysis revealed that the “optimal” concentrations of arginine varied depending on the specific gene (JAK2, STAT5, mTOR, and S6K), but within a range from 562.34 mg/L to 729.64 mg/L. Bauchart-Thevret *et al.* [35] reported that the arginine-dependent effect on cell survival and protein synthesis signaling in IPEC-J2 cells (a porcine intestinal epithelial cell line) was mediated by mTOR. The results discussed above might provide one of the explanations for the increase of casein gene expression when intracellular arginine is in an “optimal” concentration range, i.e. direct regulation of the mTOR signaling pathway by arginine.

The decrease in expression of the casein genes when arginine levels continually increased in culture medium was noteworthy. This response seems to agree to some extent with the results from Raggio *et al.* [36], i.e. the efficiency of transfer of absorbed AA into milk protein decreases markedly as protein supply increases. Clearly, further work needs to be conducted to clarify the nature of the response by mammary cells to increased AA supply.

Taken together, results of this study demonstrate for the first time that arginine plays an important role in the transcriptional regulation of casein genes and signaling pathway related genes in bovine mammary epithelial cells. Through its stimulatory effects on the expression of CSN1S1, CSN2 (in our previous work by Chen *et al.* [14]), CSN1S2, CSN3, JAK2, STAT5, mTOR and S6K, arginine supplementation at a concentration range from 369.65 mg/L to 729.64 mg/L in culture medium could stimulate casein synthesis *in vitro*. Additional experiments should be conducted to investigate the regulatory mechanism of arginine on casein expression in mammary gland of lactating cow.
